# Raman on the palm: handheld Raman spectroscopy for enhanced traceability of palm oil

**DOI:** 10.1038/s41538-025-00462-3

**Published:** 2025-06-05

**Authors:** Joe Stradling, Cassio Lima, Rudi Grosman, Igor Barsukov, Yun Xu, Ernest Teye, Chris Elliott, Howbeer Muhamadali, Royston Goodacre

**Affiliations:** 1 Centre for Metabolomics Research, Department of Biochemistry, Cell and Systems Biology, Institute of Systems, Molecular and Integrative Biology, University of Liverpool, Liverpool, UK; 2 NMR Facility, Department of Biochemistry, Cell and Systems Biology, Institute of Systems, Molecular and Integrative Biology, University of Liverpool, Liverpool, UK; 3 School of Agriculture, University of Cape Coast, Cape Coast, Central Region Ghana; 4 Institute for Global Food Security, School of Biological Sciences, Queens University Belfast, Belfast, Northern Ireland UK; 5 International Joint Research Center on Food Security (IJC-FOODSEC), Pathum Thani, Thailand

**Keywords:** Optical spectroscopy, Agriculture

## Abstract

Determining the geographic origin of palm oil in West Africa is vital for economic, environmental, and health reasons. It enhances traceability, protects local farmers, supports conservation by monitoring deforestation, and reduces food fraud, ensuring quality and regulatory compliance. Portable Raman spectroscopy offers a rapid method to identify the origin of palm oils from West Africa. Using principal component analysis (PCA), distinct clusters in scores plots were observed which reflected the geographic origin of the palm oils, with loadings from the first principal component (PC-1) highlighting β-carotene as a major source of variation among the samples. To quantify β-carotene content, a partial least squares regression (PLS-R) model was developed in coconut oil as the base oil as it is known to be β-carotene free. Once calibrated, PLS-R was used to rank the palm oil from West Africa based on their β-carotene levels. The resulting models in coconut oil demonstrated strong linearity and predictive performance, with *R*² and *Q*² values of 0.9848 and 0.9552, respectively, alongside low root mean square errors of cross-validation (0.1282 mM) and prediction (0.0747 mM); moreover, this model allows the palm oils to be ranked based on β-carotene content which was entirely reflective of the oils position in PC-1 from PCA. These findings underscore the potential of Raman spectroscopy as an effective tool for authenticating the geographic origin of palm oil from West Africa.

## Introduction

Palm oil has been used for numerous applications in a wide variety of industries including agri-food, body care and biofuels^1,2^. The palm oil industry directly employs around 6 million people globally and has increased its yield from 42 million tonnes to over 74 million tonnes between 2008 and 2020 with annual production revenues of ~$60 billion^3,4^.One of the main factors responsible for this increased production is the cost-efficiency of cultivating the oil palm plant (*Elaeis guineensis*), which is 6 to 10 times more efficient to grow per hectare than any other oilseed crop.

The recent rapid expansion of the palm oil industry has led to the increased conversion of regions of biodiversity-rich and conservation-critical areas, such as rainforests and peatlands, into large expansive plantations. Furthermore, the term ‘sustainable palm oil’ has sometimes been misused for illicit profit, with some product labels falsely claiming to contain ‘certified sustainable palm oil’ (CSPO). In response, organisations such as the Roundtable on Sustainable Palm Oil (RSPO) have been founded to create and enforce worldwide standards for sustainable palm oil. The RSPO has implemented environmental and social criteria that companies and farmers must follow to produce CSPO^5^. Beyond promoting sustainability, this global partnership supports local economies by enhancing transparency in the palm oil supply chain, allowing consumers and stakeholders to make informed decisions. Despite this initiative, concerns are mounting over increasing fraudulent activities within the palm oil industry, including adulteration, human and drug trafficking, tax evasion and illegal deforestation^6^.

A crucial aspect of adhering to sustainability standards involves identifying the geographic origin of palm oil. Previous research has explored various mass spectrometry (MS)-based techniques for determining the origin of palm oil. Ruiz-Samblás et al. used gas chromatography-mass spectrometry to analyse palm oil from Southeastern Asia, Africa, and South America, achieving success rates between 70% and 100%^7^. Another study employed gas chromatography-ion mobility spectrometry combined with chemometrics to predict the regional origin of palm oil from Malaysia^8^. High-performance liquid chromatography coupled with a charged aerosol detector and an ultraviolet detector using high and mid-level data fusion strategies, provides yet another example^9^.

Although successful, these methods require sample transportation from the palm oil source to a centralised laboratory, as well as extensive sample preparation which can be time consuming, costly and complicated. By contrast, Raman spectroscopy is an alternative tool that has the potential to be used on-site to determine geographic authenticity of palm oil in real-time. This powerful advanced analytical technique relies on the inelastic scattering of photons (Raman scattering) to determine the vibrational modes of molecules, providing a biochemical fingerprint for molecular identification^10^. The Raman phenomena has found numerous applications across a wide variety of industries and has already been applied for multiple food authenticity investigations including the evaluation of edible oils, and botanical origin identification and quantification of adulteration in honey^11,12^. Contrary to MS-based techniques Raman spectroscopy is a rapid, non-destructive, and sensitive tool that requires very little sample preparation^13^. While most studies using Raman spectroscopy rely on benchtop laboratory spectrometers, which necessitate transporting samples to a centralised facility for analysis, this technique is also available in handheld portable devices. These portable devices show promising applications in food production and quality control^14^. As shown by Hu and colleagues for the authentication of fish species, these portable devices can generate comparable levels of data acquisition to benchtop spectrometers; a testament to their potential^15^. Herein, this paper demonstrates the potential for portable Raman spectroscopy alongside chemometrics to determine the geographic origin of palm oil harvested from West Africa.

## Results and discussion

### Palm oil harvested from West Africa

**Fig. 1:**
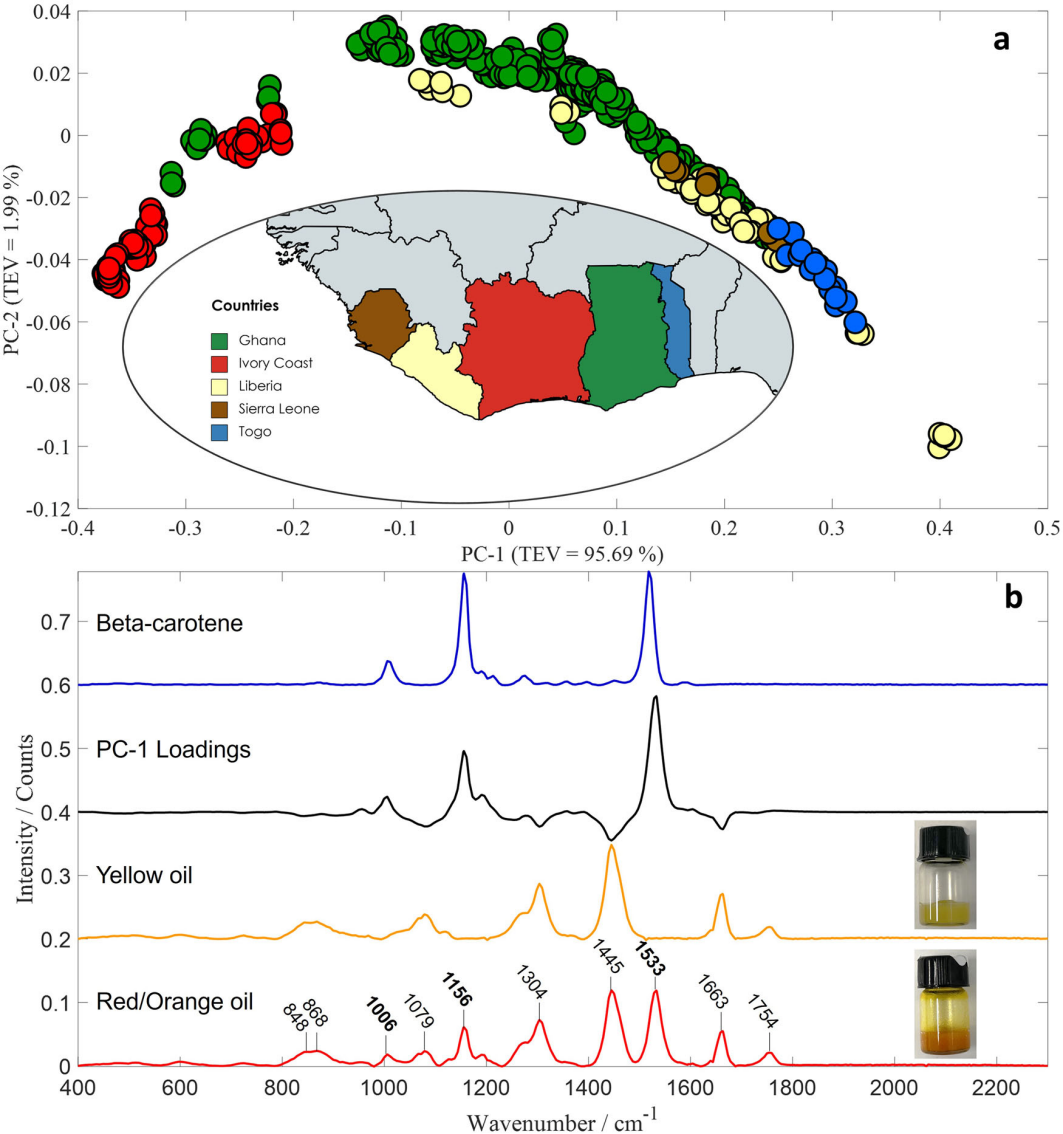
Analysis of all the palm oil samples using multivariate analysis. PCA scores plot of Raman data collected from all samples from Ghana, Ivory Coast, Liberia, Sierra Leone and Togo (**a**) as well as the associated PC-1 loadings and Raman spectra of β-carotene, the yellow oils and red/orange oils (**b**). Each sample is represented by three spectra, with each spectrum plotted as an individual score.

Raman spectra from the palm oil samples were analysed using PCA to evaluate the effectiveness of combining handheld Raman spectroscopy with chemometrics for distinguishing oils based on their geographic origins (Fig. 1 and Supplementary Fig. 2). Palm oil typically exhibits characteristic bands (see Table 1 for details) at 848 cm^−1^ and 868 cm^−1^ (C-C stretching), 1079 cm^−1^ (skeletal C-C stretching), 1304 cm^−1^ (CH₂ deformation), 1445 cm^−1^ (CH₂ scissoring), 1663 cm^−1^ (cis-C=C stretching) and 1754 cm^−1^ (C=O stretching of esters)^16^. The PC-1 loadings highlight three other Raman bands at 1006 cm^−1^, 1156 cm^−1^ and 1533 cm^−1^, which are associated with β-carotene—a natural pigment abundant in crude palm oil and responsible for its distinctive red-orange colour^17^. These bands correspond to specific molecular vibrations: C-CH₃ bonding between the carbon backbone and methyl groups, combined C=C/C-C stretching with C-H bending, and C=C stretching vibrations, respectively^18^. The presence of these bands in the PC-1 loadings indicates that variations in β-carotene content are the primary drivers of the positioning of scores along the PC-1 axis, which accounts for 95.69% of the total explained variance. Further examination of the PC-1 loadings suggests that samples located towards the positive side of the PC-1 axis possess more intense β-carotene-associated bands, whereas samples towards the negative side of the PC-1 axis show weaker or absent β-carotene signals. Therefore, the intensity of these bands - and by extension the concentration of β-carotene - provides valuable insights that can support the differentiation of oils based on their geographical origin. Moreover, previous studies investigating the classification of oil palm fruit maturity using Raman spectroscopy identified the C=C stretching vibration of β-carotene as a potential marker of fruit ripeness^19^. This implies that the positioning of a score (representing an oil sample) along the PC-1 axis may also reflect differences in fruit maturity at harvest.

**Table 1:** Raman band assignments corresponding to characteristic spectra of palm oil^19,27^

Raman shift (cm^−1^)	Assignments
848	C–C stretching
868	(C–C) stretching vibrations of amino acids
1006	C–CH_3_ in plane rocking
1079	(C–C) stretching vibration of the (CH_2_)_n_ group
1156	C–C stretching
1304	(C–H) bending twist of the CH_2_ group
1445	(C–H) scissoring of the CH_2_ group
1533	C=C stretching
1663	(C=C) and cis (C–H) groups of unsaturated fatty acids
1754	(C=O) stretching vibration of ester bond carbonyl

Given the strong influence of β-carotene content on sample differentiation, a PLS-R model was subsequently developed to predict the β-carotene concentration in each oil sample.

### Variability in β-carotene content and its impact on palm oil colour

**Fig. 2:**
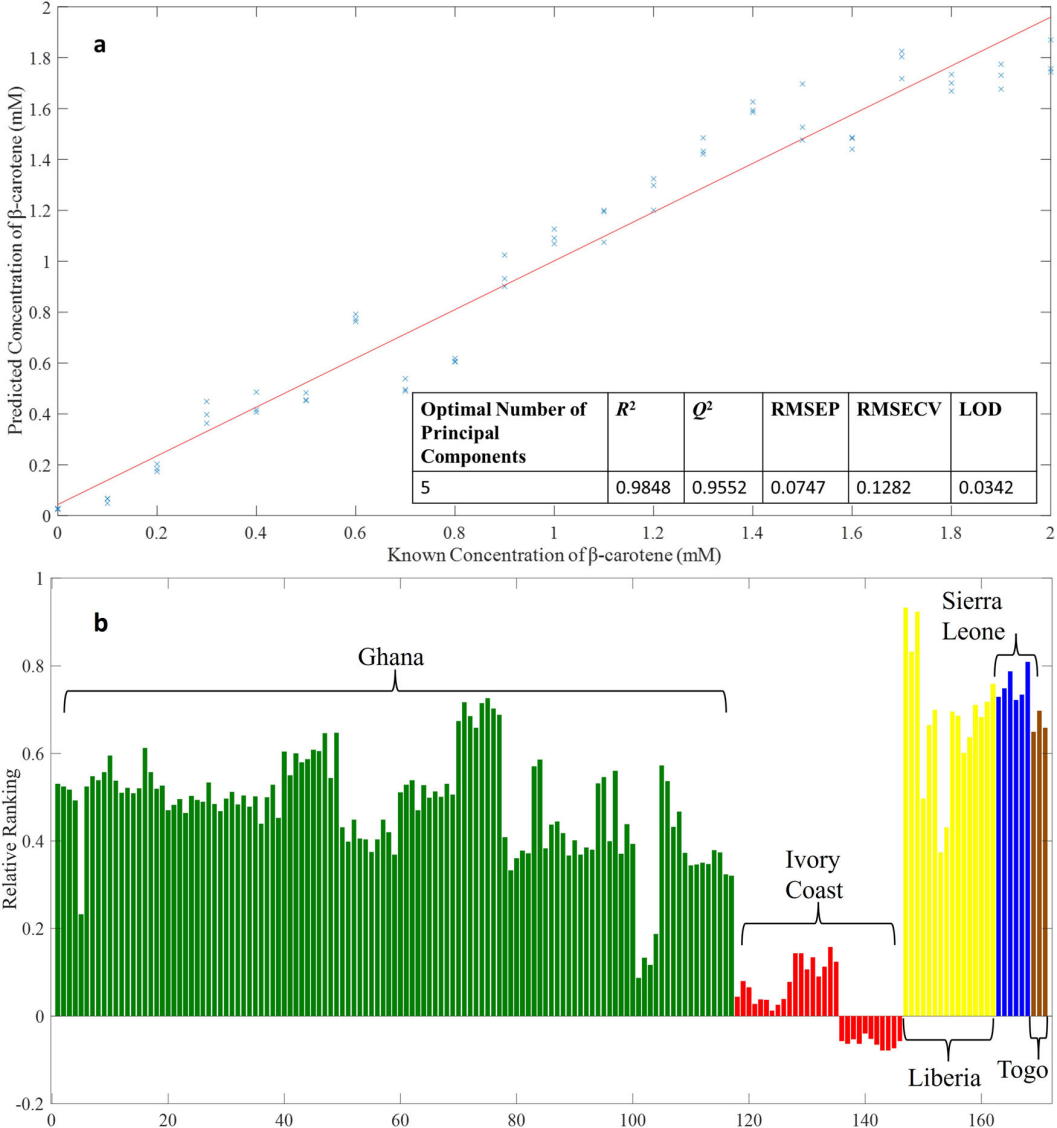
Quantitative modelling of β-carotene spiked into coconut oil to rank the palm oil samples according to β-carotene content. PLS-R prediction plot for β-carotene spiked into coconut oil across concentrations ranging from 0 to 2 mM (**a**). Three Raman spectra were recorded for each concentration and the mean value is shown. Predicted relative β-carotene levels in palm oil samples from each country based on the PLS-R model (**b**). To calculate the LoD, a PLS-R model was constructed using β-carotene spiked at concentrations between 0.1 mM to 0.5 mM, increasing in 0.1 mM increments. This concentration range was chosen because if an extended range of concentrations is used, beyond what would be seen, then the LoD generated is underestimated and thus will give an artificially optimistic result.

A PLS-R model was developed (Fig. 2a) using coconut oil samples spiked with increasing concentrations of β-carotene (ranging from 0 to 2 mM). The resulting model demonstrated strong performance, with a high coefficient of determination (*R*² = 0.9848), high predictive ability (*Q*² = 0.9552), and low error values (RMSECV = 0.1282 mM; RMSEP = 0.0747 mM). When comparing the PCA scores plot (Fig. 1a) with the predicted β-carotene concentrations for oils from different countries (Fig. 2b), clear similarities emerge. For instance, samples from the Ivory Coast cluster towards the most negative side of the PC-1 axis and show the lowest predicted β-carotene levels. In contrast, samples from Liberia are more widely distributed along the PC-1 axis, with some scores appearing at the most positive end and others clustering towards the negative side - a pattern that closely mirrors the predictive model. Interestingly, the model predicts slightly negative β-carotene levels for some oils from the Ivory Coast. However, these negative values fall within the model’s RMSECV, meaning they are effectively zero. While the presence of negative predictions highlights some limitations of the model - likely due to coconut oil being used as the base rather than β-carotene-free palm oil - the model remains a valuable tool for comparing the relative β-carotene content across different samples, and we exemplify this in Supplementary Fig. 5 by plotting some of the Raman spectra from these oils according to their β-carotene content. The calculated limit of detection (LoD) of 0.0342 mM falls outside the concentration range used for its determination. However, it remains within the range of the PLS-R model used to predict β-carotene levels. The use of coconut oil as the matrix may also contribute to the observed low LoD.

As anticipated, the oils with little to no predicted β-carotene exhibited a yellow colouration (Fig. 1b). Initially, it was suspected that these yellow oils might not be palm oil but rather palm kernel oil—a different product derived from the same fruit. Palm kernel oil naturally lacks β-carotene, has a distinct yellow appearance, and exhibits a different fatty acid composition compared to palm oil^20^. To investigate this further, NMR spectroscopy was employed to determine whether these yellow samples were indeed palm kernel oil.

### Distinguishing palm and palm kernel oils: insights from NMR

**Fig. 3:**
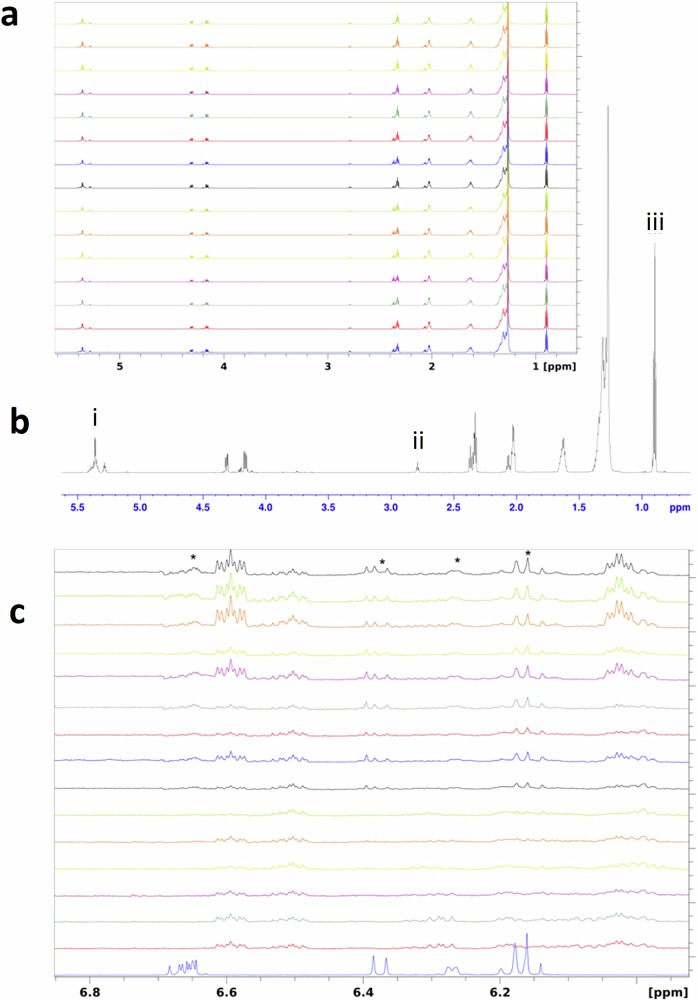
NMR analysis of palm oil composition. **a** Superposition of the 15 palm oil samples (Supplementary Table 1) selected for the analysis (spectra ordered bottom to top as in Table 2). **b** Spectrum of Sample 197 with signals used to calculate the concentrations marked; i—total double bond signal that contains contributions from oleic and linoleic fatty acid chains, ii—signal of the CH_2_ group located between the double bonds in positions 9 and 12; iii–total signal of the terminal CH_3_ groups of all fatty acid chains. **c** Superposition of the expended double bond region of β-carotene (bottom) and the palm oil samples (spectra ordered bottom to top as in Table 2).

To investigate whether the yellow oils were palm kernel oil, samples were selected from various positions along the PC-1 axis in the PCA scores plot of all oil samples (Fig. 1a). This selection included yellow-coloured samples and orange/red samples, which were subsequently analysed using NMR spectroscopy (see Supplementary Table 1 for details). Despite the differences in colour, we observed close overall similarity of the NMR spectra (Fig. 3a). To evaluate any differences in fatty acid composition we used distinct signals of the fatty acids (Fig. 3b) to estimate the content of oleic, linoleic and saturated chains in the palm oil samples. The results (Table 2) show that whilst there are slight variations in fatty acid composition between the red/orange samples and the yellow samples, the compositions of the latter align more closely with what is expected for palm oil. Thus, we can conclude that these yellow palm oils initially suspected to be kernel oil, are in fact palm oil and the colour differences must be due to something else. Comparison of the palm oil spectra with the spectrum of β-carotene (Fig. 3C) shows a lack of characteristic β-carotene signals in samples 197, 200, 159 and 168 (yellow coloured), in agreement with the Raman results. We also did not detect β-carotene signals in samples 47 and 423 which were classified as red/orange. As previously discussed, the palm oil industry is susceptible to various forms of fraud, such as the addition of synthetic dyes to enhance the oil’s red colour and falsely suggest higher nutritional value. Consequently, while samples 47 and 423 show no detectable β-carotene signals, their colours may be attributable to adulteration from colourants.

**Table 2:** Nuclear Magnetic Resonance (NMR) data for selected oil samples, detailing their sample number, colour, origin, and fatty acid profiles in comparison to typical values of palm kernel oil and palm oil

Sample Number^a^	Geographic origin	Other source information	Oleic Acid	Linoleic Acid	Total Saturated FA	Colour
197	Ivory coast	Ampremsa	39.4	13.3	47.3	yellow
200	Ivory coast	Ampremsa	41.2	14	44.8	yellow
159	Ivory coast	Sodipalm	44.4	12.5	43.1	yellow
47	Ghana	Jukwa	35.1	11.3	53.6	orange/red
168	Ivory coast	Saykro	43.3	12.2	44.5	yellow
423	Ghana	Eastern Amankum Nkwata	36.4	12.1	51.5	orange/red
441	Ghana	Pokuasi Praise Export	36.3	10.7	53	orange/red
209	Ghana	Pampromu	37.5	12.2	50.3	orange/red
400	Ghana	Eastern Asamankese	36.2	11.3	52.4	orange/red
64	Ghana	Jukwa	35.8	10.9	53.2	orange/red
392	Ghana	Eastern Bunso	38	12	50	orange/red
434	Ghana	Central Region	35.8	10.4	53.8	orange/red
187	Ghana	Volta	41.1	11.7	47.2	orange/red
229	Ghana	Ho-Dzolokpuita	39.7	12.1	48.2	orange/red
430	Togo	Togo	40.5	11.7	47.7	orange/red
**Expected Fatty Acid Composition of Palm Oil**			**~39.2**	**~10.1**	**~49.9**	**Orange/red**
**Expected Fatty Acid Composition of Palm Kernel Oil**			**~15.4**	**~2.4**	**~82.1**	**yellow**

^a^Details providing full sample numbers are available in Supplementary Table 1^28^.

### Palm oil harvested from the Ivory Coast

One limitation of this investigation was the incomplete geographical origin information for samples from Togo, Sierra Leone, and Liberia, where only the country of origin was known. In contrast, samples from Ghana and Ivory Coast included more detailed source descriptions, prompting us to focus our analysis on these samples.

**Fig. 4:**
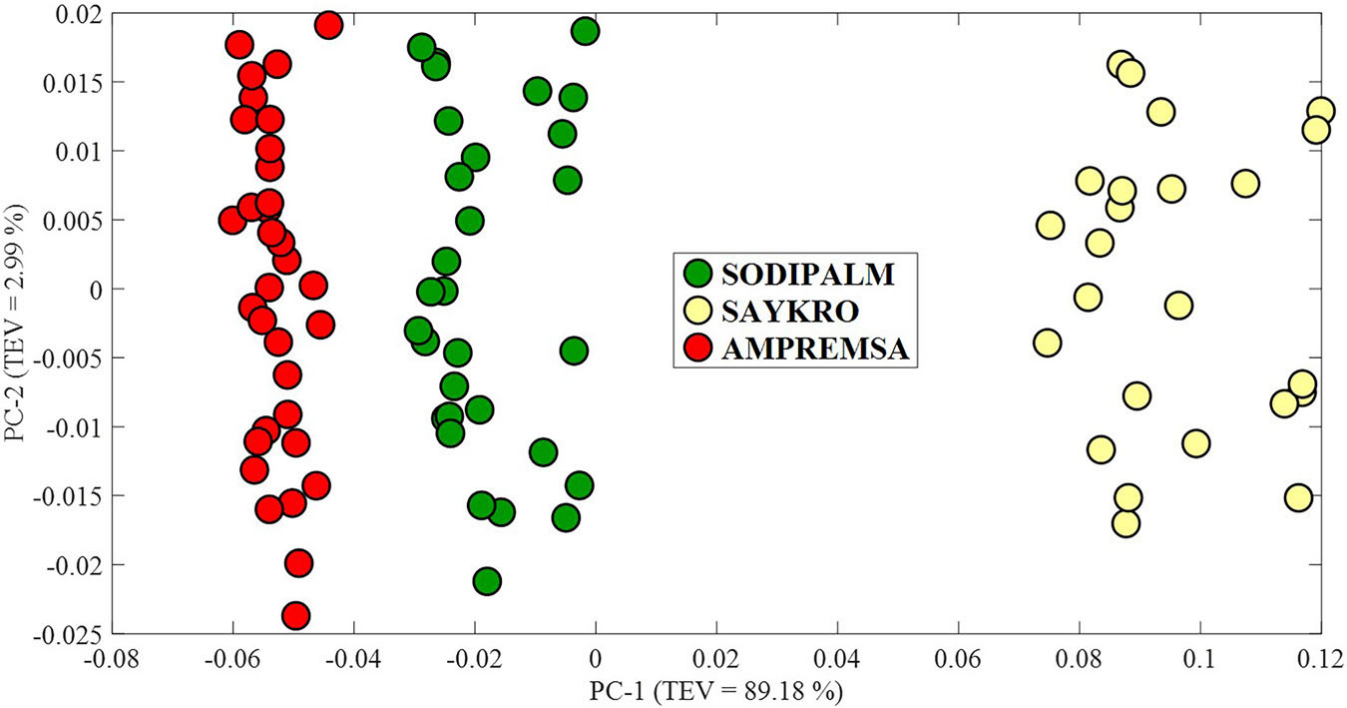
PCA scores plot of Raman spectra measured from each palm oil sample originating from various sources of the Ivory Coast. Each sample is represented by three spectra, with each spectrum plotted as an individual score. Corresponding PCA loadings plots are provided in Supplementary Fig. 3a.

Visible inspection, along with both Raman and NMR analysis, clearly showed that oils harvested from the Ivory Coast contained varying levels of β-carotene. Therefore, Raman spectra acquired from each oil harvested in the Ivory Coast were subjected to PCA to explore the use of handheld Raman spectroscopy to probe variations in the β-carotene content of the oils, and to see whether this correlated with any additional information regarding the source of the oils (Fig. 4). While oils from Sodipalm and Ampremsa show little to no evidence of bands associated with β-carotene, the oils sourced from Saykro, despite exhibiting relatively weak bands compared to those from other countries, still demonstrate sufficiently strong bands to contribute to clustering on the positive side of the PC-1 axis. Conversely, scores associated with Sodipalm and Ampremsa cluster towards the negative side of the PC-1 axis. The scores form three clear clusters which are well defined from one another, demonstrating the potential of handheld Raman spectroscopy to distinguish between palm oil from different sources.

### Palm oil harvested from Ghana

**Fig. 5:**
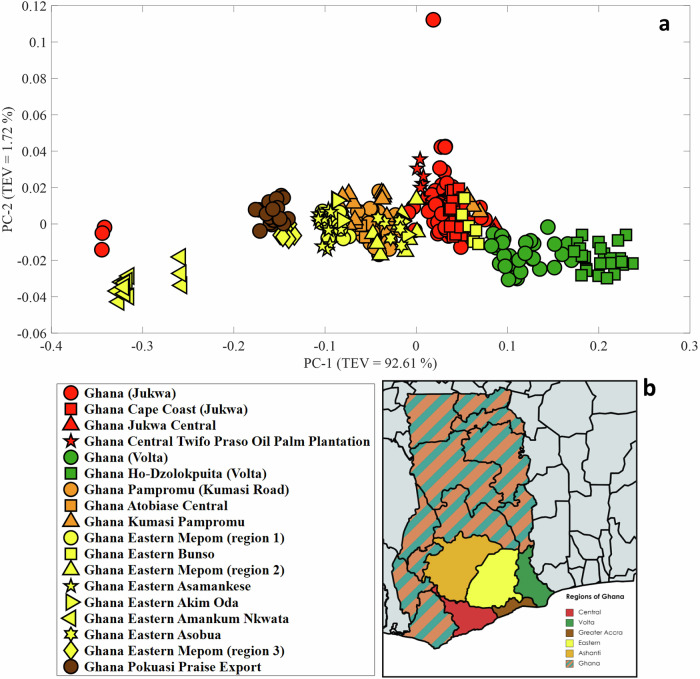
Analysis of Raman spectra from Ghana. PCA scores plot of Raman spectra for palm oil samples collected from various regions of Ghana (**a**) alongside a map highlighting their specific regions of origin (**b**). Each sample is represented by three spectra, with each spectrum plotted as an individual score. The shapes indicate the distinct sources of the oils, while the colours correspond to their respective regions of origin. Corresponding PCA loadings plots are provided in Supplementary Fig. 3b.

The oils sourced from Ghana were also subjected to PCA (Fig. 5). Unlike the oils from the Ivory Coast, the Ghanaian samples were accompanied by more detail regarding their origin, allowing us to distinguish and separate the samples based on their regions of origin. Oils from Volta and most oils from Central group on the positive side of PC-1, while oils from Ashanti, Greater Accra and Eastern group on the negative side of the PC-1 axis. Samples harvested from Volta have the most intense bands associated with β-carotene, whereas samples from Eastern Amankum Nkwata and one sample from Jukwa (Central) have the lowest intensity bands associated with β-carotene. Despite one outlier sample from Jukwa (discussed in SI and see Supplementary Fig. 4), the scores demonstrate strong clustering based on their origin, indicating that the β-carotene content or fruit maturity is generally consistent among oils from the same source. This consistency suggests a promising potential for using these characteristics as a basis for differentiation and traceability.

In Ghana, tree cover loss which is largely associated with shifting agriculture is most pronounced in regions where cocoa and palm oil farming are the major economic drivers. Among the regions analysed in this study, Ashanti (382 thousand hectares), Eastern (223 thousand hectares), and Central (215 thousand hectares) show the highest levels of tree cover loss^21^. In contrast, regions like Volta (54.5 thousand hectares) and Greater Accra (2.37 thousand hectares) experience significantly lower levels of tree cover loss. It is reasonable to suggest that areas with the most substantial tree cover loss may be experiencing the highest rates of illegal deforestation, primarily to clear land for agricultural plantations. The PCA plot of samples from Ghana indicate a relatively clear clustering of samples by their regions of origin. This clustering suggests a potential for differentiating between regions where illegal deforestation may be most intense compared to those with lower levels of illegal land clearing. With future studies involving validation in other palm oil producing areas, this finding could be useful for monitoring and targeting areas for enforcement and conservation efforts, particularly in regions heavily impacted by palm oil expansion.

In conclusion, the palm oil industry is highly vulnerable to criminal activity and unethical practices, including adulteration, mislabelling and illegal deforestation. This underscores the urgent need for robust, rapid, and effective methods to identify and trace palm oil, ensuring adherence to sustainable and ethical standards. Our study showcases β-carotene as a crucial marker for differentiating palm oil from various sources. By combining Raman spectroscopy with chemometric techniques such as PCA and PLS-R, we introduce innovative approaches for tracing palm oil back to its geographical origin. Our findings demonstrate the potential of handheld Raman devices for real-time, on-site geographical discrimination of palm oil in West Africa, enabling immediate and informed decision-making.

## Methods

### Palm oil origins

A total of 171 palm oil samples (see Supplementary Table 1) were manually harvested in 2019 from various origins: Ghana (117 samples), Togo (6 samples), Republic of Côte d’Ivoire (Ivory Coast) (29 samples), Sierra Leone (3 samples), and Liberia (16 samples). These samples were supplied by Queen’s University Belfast and were individually stored in glass vials at −20 °C prior to analysis. Supplementary Table 1 provides additional details regarding the specific geographical origins of certain oils within each country.

### Raman spectroscopy

A 1 mL aliquot of each palm oil sample was transferred into 2 mL glass vials, which were then placed in a beaker containing water and heated using a hotplate set to 50 °C; this temperature was chosen based on preliminary data as discussed in Supplementary Information (SI) and see Supplementary Fig. 1. A thermometer was placed inside the water bath and monitored regularly to ensure a consistent temperature was maintained. To prevent the samples from heating through direct contact with the bottom of the beaker—which is likely to be hotter—the vials were elevated on a custom-made plastic stand. Following heating, a single Raman spectrum was collected from each sample, with measurements repeated on two additional days to assess reproducibility, resulting in a total of three spectra per sample. Raman spectra were acquired using a CBEx handheld Raman spectrometer (Snowy Range, Laramie, Wyoming, USA) equipped with a 1064 nm laser operating at a typical power of ~30 mW. Spectral datasets were recorded in the 400–2300 cm^−1^ spectral range with an acquisition time of 10 s. The measurements were performed by placing the glass vial containing each sample into the sample holder (vial-mode). Following data collection, all spectral analysis was carried out in Matlab R2022a (The Mathworks, Natick, MA, USA). The spectra were first baseline corrected using asymmetric least squares, smoothed with a Savitzky–Golay filter with a 2nd order polynomial and a window width of 11 points and vector normalised^22,23^. For all Partial Least Squares Regression (PLS-R) analysis, spectra were vector-normalised only, with no further pre-processing which could lead to overfitting of the data. Principal Component Analysis (PCA) was applied to visualise the relationship between the Raman data and PLS-R was employed to model the contents of β-carotene in palm oil quantitatively^24,25^. To construct these PLS-R models, β-carotene was spiked into coconut oil at concentrations ranging from 0 to 2 mM, increasing in 0.1 mM increments. This concentration range was selected as it encompasses the typical β-carotene levels reported in palm oil in literature^26^. Coconut oil was selected as a matrix due to its compositional similarity to palm oil and the absence of inherent β-carotene. Three replicate samples were prepared at each concentration, and Raman spectra were collected for all samples. The spectra of the coconut oil spiked with varying levels of β-carotene served as the training dataset, while the Raman spectra obtained from the palm oil samples were used as the test dataset.

### Nuclear magnetic resonance (NMR) spectroscopy

Samples for NMR spectroscopy were prepared by mixing 20 μL of sample with 700 μL CDCl_3_ followed by transferring 600 μL of this mixture to 5 mm, 4-inch NMR Tubes (Bruker). Samples were stored in a fridge at 4 °C until acquisition. NMR spectra were acquired at 15 °C using Bruker Neo 800 MHz spectrometer equipped with TCI CryoProbe. One-dimensional ^1^H NMR spectra were recorded using a single-pulse experiment (zg, Bruker) with a 7.3 ppm spectral width, offset of 3.2 ppm, acquisition time 2.3 s and a 4.8 s inter-scan delay. Spectra were processed with TopSpin 4.09 and analysed using in-house software. Chemical shifts were referenced to the internal TMS signal at 0 ppm. Single time zero filling and line broadening of 0.5 Hz was applied before processing. Spectra were phase and baseline corrected using absn command of TopSpin. Local base-line correction was used for the signal integration. Signals of the main components were assigned by comparing them with the signals of in-house standards measured under the same conditions.

## Supplementary information

Raman on the Palm SI Final

## Data Availability

The data are available on request. Data processing algorithms are available via: https://github.com/Biospec/.
