# Supplementary material for: Raman on the palm: handheld Raman spectroscopy for enhanced traceability of palm oil
Source: NPJ Sci Food. 2025 Jun 5;9:95. doi: 10.1038/s41538-025-00462-3 (PMC12141510; doi:10.1038/s41538-025-00462-3)
Supplement: Supplementary file 1 — Raman on the Palm SI Final [file 41538_2025_462_MOESM1_ESM.pdf]

Raman on the Palm: Handheld Raman Spectroscopy for Enhanced Traceability of Palm Oil

Joe Stradling[1], Cassio Lima[1], Rudi Grosman[2], Igor Barsukov[2], Yun Xu[1], Ernest Teye[3], Chris Elliott[4,5], Howbeer Muhamadali[1], and Royston Goodacre[1]*

[1] *Centre for Metabolomics Research, Department of Biochemistry, Cell and Systems Biology, Institute of Systems, Molecular and Integrative Biology, University of Liverpool, Liverpool L69 7ZB, United Kingdom*

[2] *NMR facility, Department of Biochemistry, Cell and Systems Biology, Institute of Systems, Molecular and Integrative Biology, University of Liverpool, Liverpool L69 7ZB, United Kingdom*

[3] *School of Agriculture, University of Cape Coast, Cape Coast, Central Region, Ghana*

[4] *Institute for Global Food Security, School of Biological Sciences, Queens University Belfast, 19 Chlorine Gardens, Belfast BT9 5DL, Northern Ireland, UK*

[5] *International Joint Research Center on Food Security (IJC-FOODSEC), 111 Thailand Science Park, 16 Phahonyothin Road, Pathum Thani, 12120, Thailand*

*correspondence to: roy.goodacre@liverpool.ac.uk

Supplementary information

Table S1. Palm oil samples with original labels, sources, and NMR analysis selection.

Sample number	Country	Local Origin$	Samples used for NMR analysis (*)
CPO-0043	Ghana	Jukwa	
CPO-0044	Ghana	Jukwa	
CPO-0045	Ghana	Jukwa	
CPO-0046	Ghana	Jukwa	
CPO-0047	Ghana	Jukwa	*
CPO-0048	Ghana	Jukwa	
CPO-0049	Ghana	Jukwa	
CPO-0050	Ghana	Jukwa	
CPO-0051	Ghana	Jukwa	
CPO-0052	Ghana	Jukwa	
CPO-0053	Ghana	Jukwa	
CPO-0054	Ghana	Jukwa	
CPO-0055	Ghana	Jukwa	
CPO-0056	Ghana	Jukwa	
CPO-0057	Ghana	Jukwa	
CPO-0058	Ghana	Jukwa	
CPO-0059	Ghana	Jukwa	
CPO-0060	Ghana	Jukwa	
CPO-0061	Ghana	Jukwa	
CPO-0062	Ghana	Jukwa	
CPO-0063	Ghana	Jukwa	
CPO-0064	Ghana	Jukwa)	*
CPO-0065	Ghana	Jukwa	
CPO-0066	Ghana	Jukwa	

CPO-0067	Ghana	Jukwa	
CPO-0068	Ghana	Jukwa	
CPO-0069	Ghana	Jukwa	
CPO-0070	Ghana	Jukwa	
CPO-0071	Ghana	Jukwa	
CPO-0072	Ghana	Jukwa	
CPO-0073	Ghana	Jukwa	
CPO-0074	Ghana	Jukwa	
CPO-0075	Ghana	Jukwa	
CPO-0076	Ghana	Jukwa	
CPO-0077	Ghana	Jukwa	
CPO-0078	Ghana	Jukwa	
CPO-0079	Ghana	Jukwa	
CPO-0080	Ghana	Jukwa	
CPO-0081	Ghana	Jukwa	
CPO-0136	Liberia	-	
CPO-0137	Liberia	-	
CPO-0138	Liberia	-	
CPO-0139	Liberia	-	
CPO-0140	Liberia	-	
CPO-0141	Liberia	-	
CPO-0142	Liberia	-	
CPO-0143	Liberia	-	
CPO-0144	Liberia	-	
CPO-0145	Liberia	-	
CPO-0146	Liberia	-	
CPO-0147	Liberia	-	
CPO-0148	Liberia	-	
CPO-0149	Liberia	-	
CPO-0150	Liberia	-	
CPO-0151	Liberia	-	
CPO-0154	Ivory Coast	SODIPALM - Cote d'Ivoire	
CPO-0155	Ivory Coast	SODIPALM - Cote d'Ivoire	
CPO-0156	Ivory Coast	SODIPALM - Cote d'Ivoire	
CPO-0157	Ivory Coast	SODIPALM - Cote d'Ivoire	
CPO-0158	Ivory Coast	SODIPALM - Cote d'Ivoire	
CPO-0159	Ivory Coast	SODIPALM - Cote d'Ivoire	*
CPO-0160	Ivory Coast	SODIPALM - Cote d'Ivoire	
CPO-0161	Ivory Coast	SODIPALM - Cote d'Ivoire	
CPO-0162	Ivory Coast	SODIPALM - Cote d'Ivoire	
CPO-0163	Ivory Coast	SODIPALM - Cote d'Ivoire	
CPO-0164	Ivory Coast	SAYKRO - Cote d'Ivoire	
CPO-0165	Ivory Coast	SAYKRO - Cote d'Ivoire	
CPO-0166	Ivory Coast	SAYKRO - Cote d'Ivoire	
CPO-0167	Ivory Coast	SAYKRO - Cote d'Ivoire	
CPO-0168	Ivory Coast	SAYKRO - Cote d'Ivoire	*

CPO-0169	Ivory Coast	SAYKRO - Cote d'Ivoire	
CPO-0170	Ivory Coast	SAYKRO - Cote d'Ivoire	
CPO-0171	Ivory Coast	SAYKRO - Cote d'Ivoire	
CPO-0182	Ghana	Volta	
CPO-0183	Ghana	Volta	
CPO-0184	Ghana	Volta	
CPO-0185	Ghana	Volta	
CPO-0186	Ghana	Volta	
CPO-0187	Ghana	Volta	*
CPO-0188	Ghana	Volta	
CPO-0189	Ghana	Volta	
CPO-0190	Ghana	Volta	
CPO-0191	Ghana	Volta	
CPO-0193	Ivory Coast	AMPREMSA - Cote D'Ivoire	
CPO-0194	Ivory Coast	AMPREMSA - Cote D'Ivoire	
CPO-0195	Ivory Coast	AMPREMSA - Cote D'Ivoire	
CPO-0196	Ivory Coast	AMPREMSA - Cote D'Ivoire	
CPO-0197	Ivory Coast	AMPREMSA - Cote D'Ivoire	*
CPO-0198	Ivory Coast	AMPREMSA - Cote D'Ivoire	
CPO-0199	Ivory Coast	AMPREMSA - Cote D'Ivoire	
CPO-0200	Ivory Coast	AMPREMSA - Cote D'Ivoire	*
CPO-0201	Ivory Coast	AMPREMSA - Cote D'Ivoire	
CPO-0202	Ivory Coast	AMPREMSA - Cote D'Ivoire	
CPO-0203	Ivory Coast	AMPREMSA - Cote D'Ivoire	
CPO-0205	Ghana	PAMPROMU - Kumasi Road	
CPO-0206	Ghana	PAMPROMU - Kumasi Road	
CPO-0207	Ghana	PAMPROMU - Kumasi Road	
CPO-0208	Ghana	PAMPROMU - Kumasi Road	
CPO-0209	Ghana	PAMPROMU - Kumasi Road	*
CPO-0210	Ghana	PAMPROMU - Kumasi Road	
CPO-0211	Ghana	PAMPROMU - Kumasi Road	
CPO-0212	Ghana	PAMPROMU - Kumasi Road	
CPO-0213	Ghana	PAMPROMU - Kumasi Road	
CPO-0214	Ghana	PAMPROMU - Kumasi Road	
CPO-0215	Ghana	Cape Coast - Jukwa	
CPO-0216	Ghana	Cape Coast - Jukwa	
CPO-0217	Ghana	Cape Coast - Jukwa	
CPO-0218	Ghana	Cape Coast - Jukwa	
CPO-0219	Ghana	Cape Coast - Jukwa	
CPO-0220	Ghana	Cape Coast - Jukwa	
CPO-0221	Ghana	Cape Coast - Jukwa	
CPO-0222	Ghana	Cape Coast - Jukwa	
CPO-0223	Ghana	Cape Coast - Jukwa	
CPO-0224	Ghana	Cape Coast - Jukwa	
CPO-0225	Ghana	Ho-Dzolokpuita (Volta region)	
CPO-0228	Ghana	Ho-Dzolokpuita (Volta region)	

CPO-0229	Ghana	Ho-Dzolokpuita (Volta region)	*
CPO-0230	Ghana	Ho-Dzolokpuita (Volta region)	
CPO-0231	Ghana	Ho-Dzolokpuita (Volta region)	
CPO-0232	Ghana	Ho-Dzolokpuita (Volta region)	
CPO-0233	Ghana	Ho-Dzolokpuita (Volta region)	
CPO-0234	Ghana	Ho-Dzolokpuita (Volta region)	
CPO-0387	Ghana	EASTERN MEPOM2	
CPO-0388	Ghana	EASTERN MEPOM4	
CPO-0389	Ghana	EASTERN MEPOM5	
CPO-0390	Ghana	EASTERN MEPOM6	
CPO-0391	Ghana	EASTERN MEPOM7	
CPO-0392	Ghana	EASTERN BUNSO	*
CPO-0393	Ghana	EASTERN BUNSO	
CPO-0394	Ghana	ATOBIASE CENTRAL	
CPO-0395	Ghana	EASTERN MEPOM	
CPO-0396	Ghana	EASTERN MEPOM	
CPO-0397	Ghana	EASTERN MEPOM	
CPO-0398	Ghana	EASTERN ASAMANKESE	
CPO-0399	Ghana	EASTERN ASAMANKESE	
CPO-0400	Ghana	EASTERN ASAMANKESE	*
CPO-0401	Ghana	EASTERN ASAMANKESE	
CPO-0402	Ghana	EASTERN AKIM ODA	
CPO-0403	Ghana	EASTERN AKIM ODA	
CPO-0404	Ghana	JUKWA CENTRAL	
CPO-0410	Ghana	KUMASI PAMPROM	
CPO-0411	Ghana	KUMASI PAMPROM	
CPO-0412	Ghana	KUMASI PAMPROM	
CPO-0413	Ghana	KUMASI PAMPROM	
CPO-0414	Ghana	KUMASI PAMPROM	
CPO-0415	Sierra Leone	-	
CPO-0416	Sierra Leone	-	
CPO-0417	Sierra Leone	-	
CPO-0422	Ghana	EASTERN AMANKUM NKWATA	
CPO-0423	Ghana	EASTERN AMANKUM NKWATA	*
CPO-0424	Ghana	EASTERN AMANKUM NKWATA	
CPO-0425	Ghana	EASTERN AMANKUM NKWATA	
CPO-0428	Togo	-	
CPO-0429	Togo	-	
CPO-0430	Togo	-	*
CPO-0431	Togo	-	
CPO-0432	Togo	-	
CPO-0433	Togo	-	
CPO-0434	Ghana	CENTRAL REGION TWIFO PRASO OIL PALM PLANTATION	*
CPO-0435	Ghana	CENTRAL REGION TWIFO PRASO OIL PALM PLANTATION	
CPO-0436	Ghana	EASTERN ASOBUA	

CPO-0437	Ghana	EASTERN ASOBUA	
CPO-0438	Ghana	POKUASI PRAISE EXPORT	
CPO-0439	Ghana	POKUASI PRAISE EXPORT	
CPO-0440	Ghana	POKUASI PRAISE EXPORT	
CPO-0441	Ghana	POKUASI PRAISE EXPORT	*
CPO-0442	Ghana	POKUASI PRAISE EXPORT	
CPO-0443	Ghana	POKUASI PRAISE EXPORT	
CPO-0444	Ghana	POKUASI PRAISE EXPORT	
CPO-0445	Ghana	EASTERN MEPOM5	
CPO-0446	Ghana	EASTERN MEPOM6	

$ Some of the palm oil samples were sourced from local processors, and we used their locations as identifiers since they do not have company/plantation names.

Preliminary Data to establish optimum collection of Raman spectra from Palm Oil

In liquids, where molecules can rotate and vibrate freely, Raman-active vibrational modes are often more clearly represented than in solids, where molecular motion is restricted. To ensure the oils were fully in their liquid state, they were heated prior to measurement. Palm oil has a melting point between 27 and 42.5 °C, so a temperature of 50 °C was selected to guarantee complete melting and to prevent any solid residues that could interfere with the Raman signal. The oils were heated for 10 minutes, then allowed to cool for 5 minutes before Raman measurements were taken at room temperature. Fig. S1 shows Raman spectra collected from palm oil after heating and during cooling over a period of up to 3 hours.

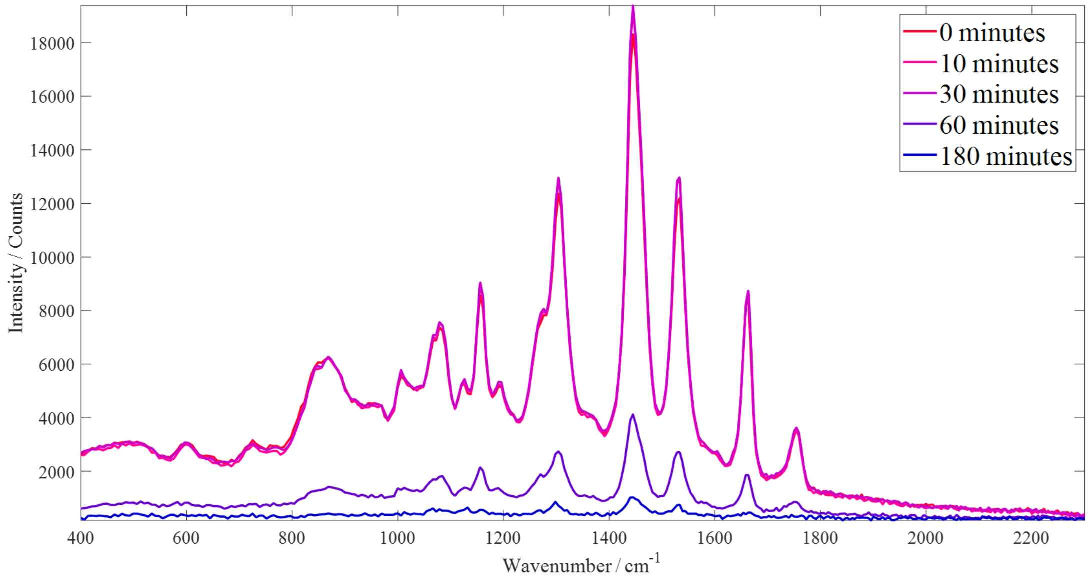

Fig. S1 Raman spectra of palm oil recorded at various cooling times over a 3-hour period after heating to 50 °C for 10 min.

Additional discussion on PCA scores and loadings plots from Raman data

There is some degree of clustering along the PC-2 axis when comparing the spectra collected from each country (Fig. S2a). The PC-2 loadings (Fig. S2b) is largely dominated by three peaks at 1304 cm^{-1}, 1445 cm^{-1}, and 1533 cm^{-1}. The first two bands are attributed to skeletal vibrations, likely associated with fatty acids, while the third band corresponds to β-carotene. Although clustering between oils based on fatty acid differences is observable, closer examination of the PCA and PC-2 loadings for samples from just the Ivory Coast (Fig. S3a) and just Ghana (Fig. S3b) reveal very little significant information. Therefore, the primary focus of the PCA in this study is placed on β-carotene variation as captured by the PC-1 loadings.

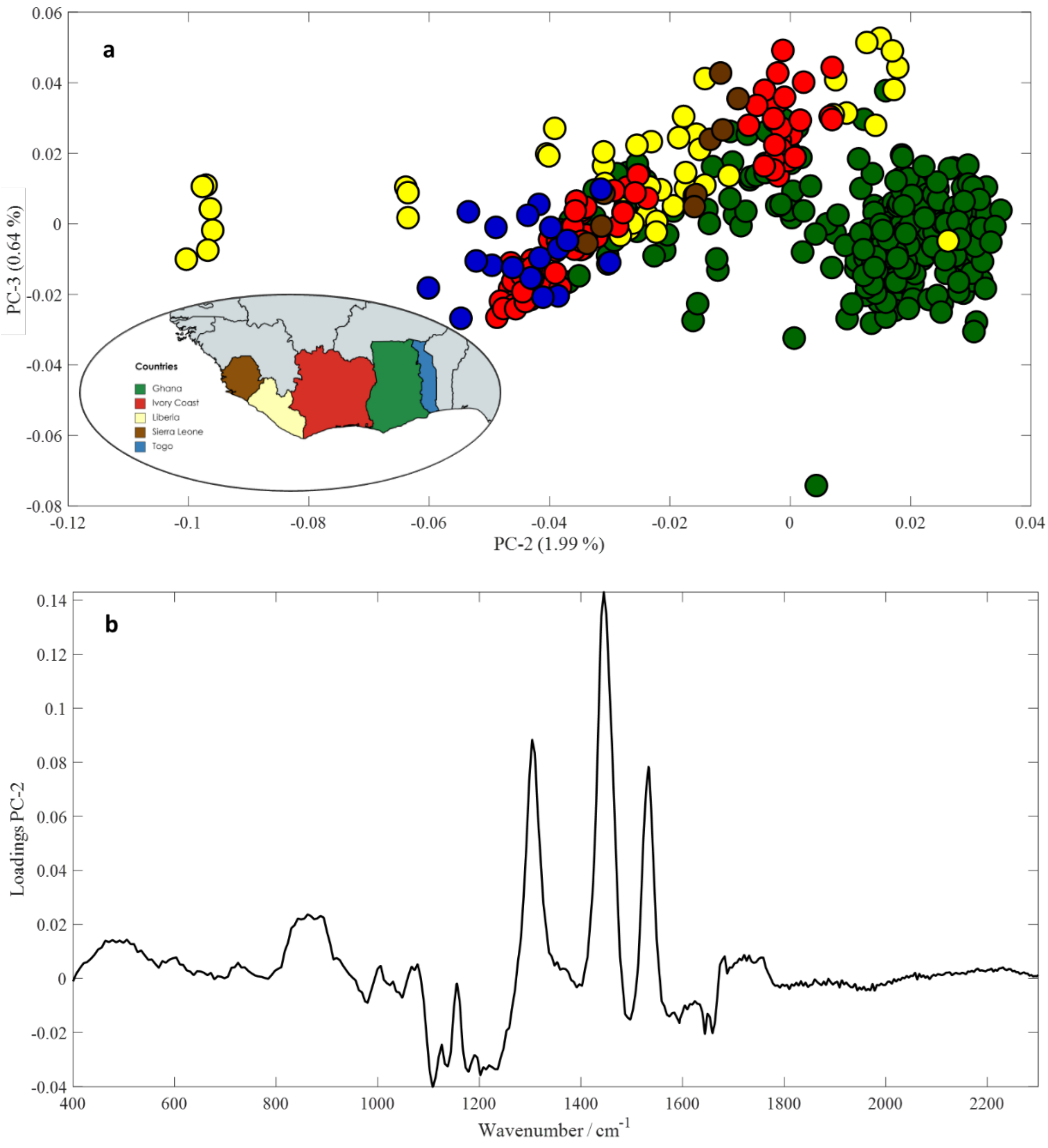

Fig. S2 PCA scores plot of Raman data collected from all samples from Ghana, Ivory Coast, Liberia, Sierra Leone and Togo using PC-2 and PC-3 (a) as well as the associated PC-2 loadings (b). Each sample is represented by three spectra, with each spectrum plotted as an individual score.

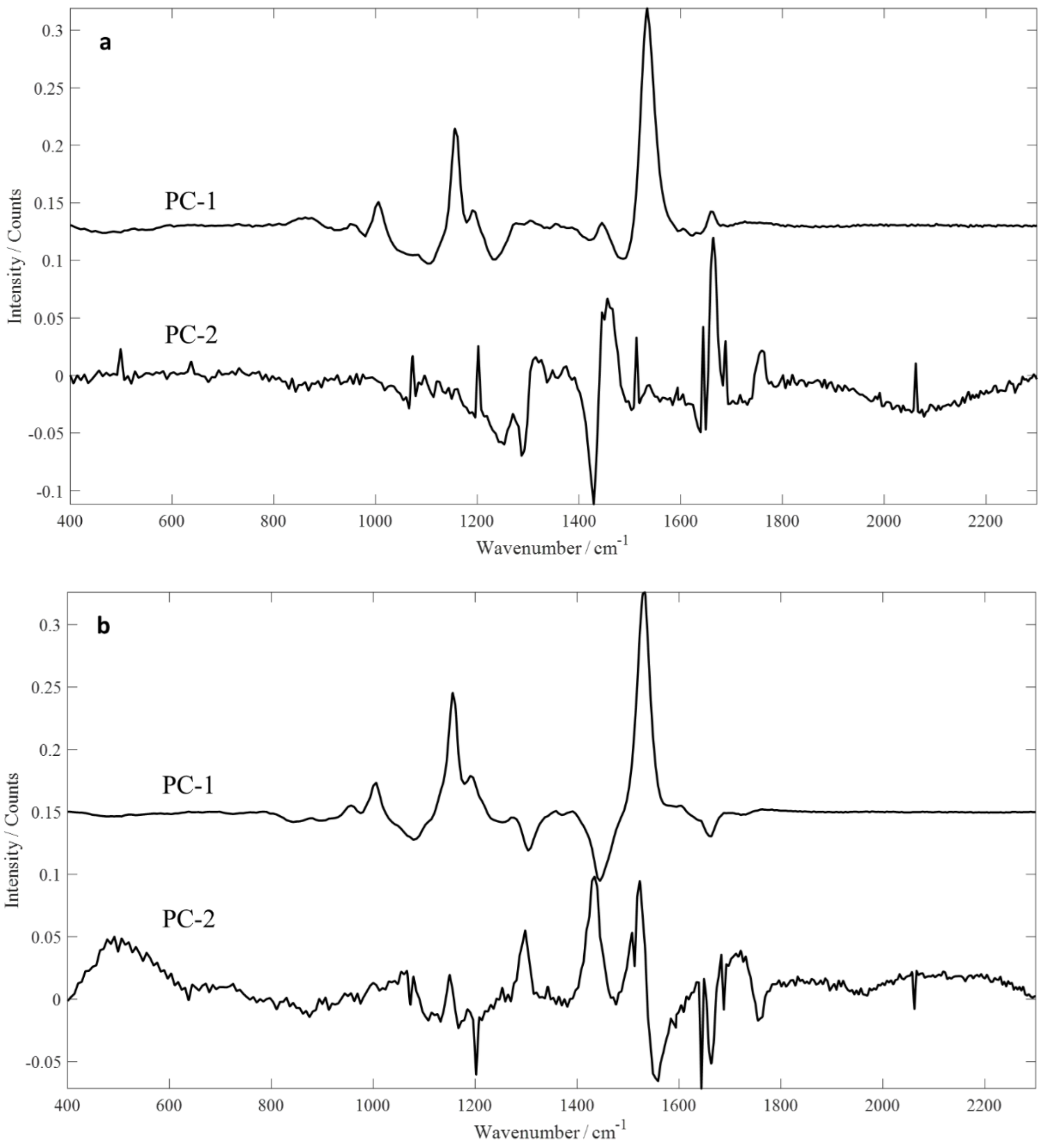

Fig. S3 The PC-1 and PC-2 loadings of the PCA of samples from the Ivory Coast (a) and Ghana (b).

A single outlier is seen from one of the replicates from a palm oil from Jukwa

By comparing the normalised spectrum of the outlier sample (a single red score representing a sample from Jukwa (Fig. 5a)) with the spectrum of the same sample collected on a different day (Fig. S4), it is evident that the signal intensity has decreased and the baseline in the lower wavenumber region is elevated. These factors explain why this spectrum appears as an outlier in Fig. 5a. A potential cause for this could be incomplete melting of the oil, although there is no direct evidence of this.

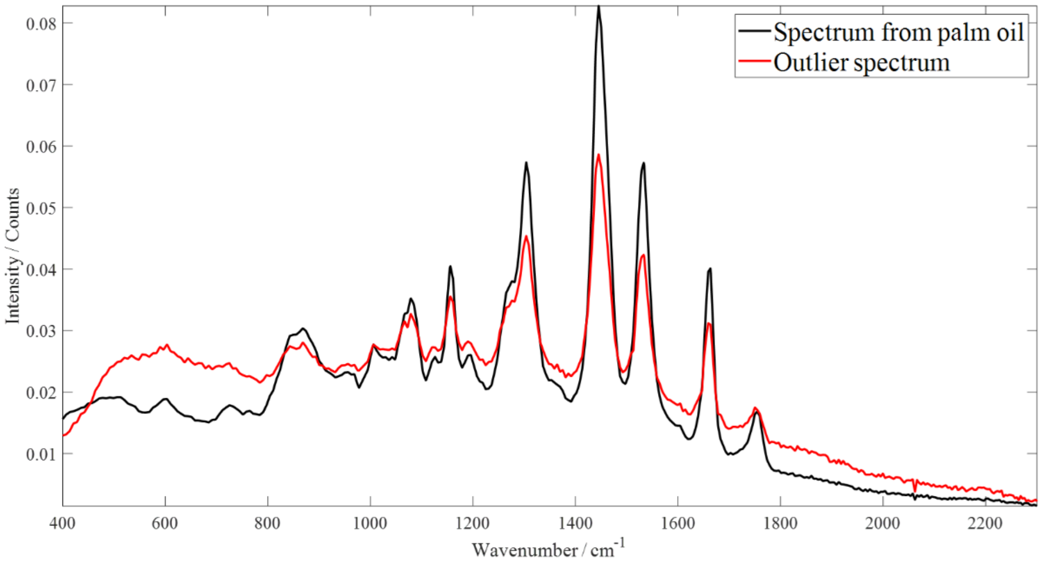

Fig. S4 The raw spectrum of the outlier score in the PCA of samples from Ghana (Fig. 5) compared to the spectrum of the same sample measured on a different day. The spectra have been normalised to enhance visual differences.

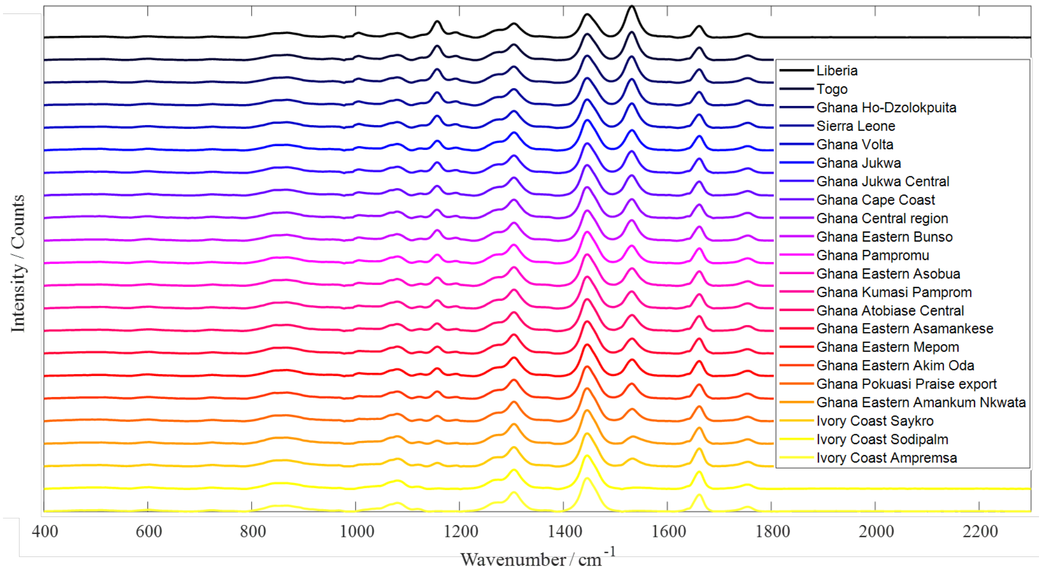

Fig. S5 Pre-processed and averaged Raman spectra of palm oil samples, grouped by region of origin and ordered by the intensity of β-carotene-associated bands.
